# The Permanent Memorial

**Published:** 1920-05-08

**Authors:** Arthur Stanley


### THE PERMANENT MEMORIAL.

### By the Hon. Sir Arthur Stanley, G.B.E., C.B.

By the lamented death of Sir Henry Burdett the hospitals and kindred institutions throughout the whole country have suffered an irreparable loss. Sir Henry Burdett was a man of lofty ideals and of most splendid enthusiasm. He saw far ahead, and this is what gave special weight and importance to his advice and practical assistance. When once he had foreseen the needs of an institution, or a body of institutions such as the hospitals of this country, he formed in his own mind an absolutely clear idea of the course to be pursued, and proceeded to work to that idea with unwearying energy and the ability which came from complete knowledge of his subject.

He was a pioneer in seeking for the advancement of hospital administration and in securing and co-ordinating the interests of the public in hospital matters. It must always be remembered that to him was due the idea of standardising accounts, which has been adopted by King Edward's Hospital Fund in the case of the London hospitals, and has been found to work so well.

At the time of his death he was most keenly interested in dealing with the crisis that has arisen in the hospital world. It was only a short time ago that I saw him and heard from his own lips how ardently he hoped to preserve the voluntary nature of the hospitals of the country and the plans which he had formed to this end. Although he was already then suffering in health, he had all the enthusiasm of his earlier years, and there is no doubt that had he been spared he would once more have helped the hospitals to surmount their difficulties as he has done so often in the past.

To no institution was he a better friend than to the College of Nursing. From the first conception of the idea of the College he was always ready with assistance and advice. On many occasions he was ready also with criticism, but it was always helpful criticism of a constructive nature.

His great life-work in connection with nurses was undoubtedly the establishment of the Royal National Pension Fund for Nurses and Hospital Officials. At the outset he had many difficulties and much opposition to overcome, but he triumphed over them all and has left as a permanent memorial to his name the Pension Fund for Nurses, which has made it possible, even in the days when nurses were paid such inadequate salaries, for them to make some provision for their old age.

His pen was always at the service of nurses and the Nursing Profession, and in all his views, which he advocated with great force and ability, he worked with a single eye to the advancement of the Nursing Profession and to the material welfare of the nurses themselves.

He was a many-sided man, and all sections of the community, especially those dealing with humanitarian work, have cause to remember his name with reverence and affection. His place will be impossible to fill, but his memory can and will be preserved as a bright example of what can be done by a man who, regardless of himself, sets out to do good to his fellow men.

